# Two Glycosylation Sites in H5N1 Influenza Virus Hemagglutinin That Affect Binding Preference by Computer-Based Analysis

**DOI:** 10.1371/journal.pone.0038794

**Published:** 2012-06-14

**Authors:** Wentian Chen, Shisheng Sun, Zheng Li

**Affiliations:** 1 Laboratory for Functional Glycomics, College of Life Sciences, Northwest University, Xi’an, People’s Republic of China; 2 Department of Pathology, Clinical Chemistry Division, Johns Hopkins University, Baltimore, United States of America; University of Ottawa, Canada

## Abstract

Increasing numbers of H5N1 influenza viruses (IVs) are responsible for human deaths, especially in North Africa and Southeast Asian. The binding of hemagglutinin (HA) on the viral surface to host sialic acid (SA) receptors is a requisite step in the infection process. Phylogenetic analysis reveals that H5N1 viruses can be divided into 10 clades based on their HA sequences, with most human IVs centered from clade 1 and clade 2.1 to clade 2.3. Protein sequence alignment in various clades indicates the high conservation in the receptor-binding domains (RBDs) is essential for binding with the SA receptor. Two glycosylation sites, 158N and 169N, also participate in receptor recognition. In the present work, we attempted to construct a serial H5N1 HA models including diverse glycosylated HAs to simulate the binding process with various SA receptors *in silico*. As the SA-α-2,3-Gal and SA-α-2,6-Gal receptor adopted two distinctive topologies, straight and fishhook-like, respectively, the presence of N-glycans at 158N would decrease the affinity of HA for all of the receptors, particularly SA-α-2,6-Gal analogs. The steric clashes of the huge glycans shown at another glycosylation site, 169N, located on an adjacent HA monomer, would be more effective in preventing the binding of SA-α-2,3-Gal analogs.

## Introduction

The influenza viruses (IVs) have had an important effect on human history. Three influenza A pandemics have taken place in 1918–1919 (H1N1), 1957 (H2N2) and 1968 (H3N2), which killed up to 50 million people all over the world. According to phylogenetic analysis, IVs originated in waterfowl; due to antigenic mutations, including antigenic drift and antigenic shift, the mutation in the receptor-binding domains (RBDs) could result in a human pandemic similar to H1N1 [1]. Since the last century, there are increasing numbers of reports about avian IVs, such as the H5N1, H7N7 and H9N2 virus, that have infected humans [2], [3]. As of 02 May 2012, 603 confirmed human cases with avian influenza H5N1 virus were reported to the WHO since 2003; of these patients, 356 died (fatality rate: 59.03%) [4].

The recognition, attachment and infection of IVs require the participation of a kind of enveloped glycoprotein, named hemagglutinin (HA), which is a homotrimeric transmembrane protein. Three RBDs at the tip of HA binding to the host sialoglycans are essential for endocytosis, each monomer can be cleaved by proteases in phagosome into a globular head and a stem region, known as HA1 and HA2, respectively [5], [6]. The whole HA unfold and expose their interior HA2 in the acid environment, then the fusion peptides in HA2 insert themselves into the host membrane and lock the membrane bilayers. Finally, uncharacterized changes to its conformation pull the two membranes together [7], [8].

As the key role in the infection process, HA is thought to be crucial in host switching. HA interacts with the distal sialic acid (SA) and the penultimate β-D-galactose (Gal) on the host glycans. Commonly, the human IVs prefer the SA-α-2,6-Gal terminal glycan, whereas the avian IVs prefer SA-α-2,3-Gal [9]. SA-α-2,6-Gal is the characteristic glycan pattern in the upper respiratory airway of humans, but a small amount of SA-α-2,3-Gal also appears in the lower respiratory airway and breast milk in humans [10], [11]. To cross the species barrier and result in the human-to-human infections, HA must change its receptor-binding preference from SA-α-2,3-Gal to SA-α-2,6-Gal [12].

Studies of H5N1 virus demonstrate that receptor recognition occurs in the RBD. The RBD consists of three secondary structure elements and four conserved residues: Helix190, Loop130 and Loop220 combined with 98Tyr, 153Trp, 183His, and 195Tyr [13]. The Q226L and G228S mutations in HA of H3N2 and H2N2 virus, correlated with the host switching from SA-α-2,3-Gal to SA-α-2,6-Gal receptor specificity [14]. Actually, as the pocket is shallow, the mutation of one or two residues in the RBD would be responsible for host switching. The S227N mutation resulted in binding to SA-α-2,3-Gal receptors with lower affinity, which caused several human infections in Hong Kong and Egypt [15]. The combination of the Q226L and G228S mutations would be more efficient in binding SA-α-2,6-Gal, though there is no such mutation in H5N1 [16].

Glycosylation plays an important role in the viral life cycle. Among the different subtypes of IV HA, there are extensive variations in the glycosylation sites of the head region, whereas the stem region are more conserved [17]. Glycans near the antigenic epitopes interfere with antibody recognition to escape immune pressure from hosts, and those near HA0 cleavage site protect their infectivity [18], [19]. The gain or loss of glycosylation sites near the RBD can also affect viral receptor recognition [20], [21]. Sun *et al.* describe several alternative models of glycosylation sites that affect the influenza virus, such as increasing the number of glycosylation sites and changing their positional conversion. One conspicuous feature is that, when a new human epidemic virus emerges, the virus would reveal a high affinity to the host target. As co-evolution occurs with the host, the virulence of the virus would decrease, accompanied by a lower affinity and increasing numbers of glycosylation site [22].

The N-glycan synthesis of viral glycoprotein depended on the host cell. Non-glycosylated HA can be obtained by expression in *Escherichia coli* [23]. HA expressed in insect cells, such as S2 cells, differs from the HA in mammalian cells, with heterogeneous mannose-type glycans in the N-glycan terminus [24]. Additionally, high-mannose-type glycans can be produced in human cells deficient in a glycosyltransferase named N-acetylglucosaminyltransferase I (GnTI-HEK293 cells) [25]. Wang *et al.* detect the HAs with gradually increasing glycosylation by glycan microarrays and find that their binding ability is not only related to different SA candidates but also to the glycosylation around the RBD. The truncation of the N-glycans on HA increased its binding affinity while decreasing its specificity toward disparate SA receptors [26].

To further explore the binding affinity between HA and SA receptors, computational methods were adopted [12], [13], [27]. Commonly, researchers focused on the interactions between HA and different SA receptors, with less consideration of the N-glycosylation on HA. In the present study, the contribution of the glycosylation of HA to host recognition was analyzed by available H5N1 HA sequences and crystal structures. A phylogenetic analysis was used to investigate the key amino-acid residues and glycosylation sites in the RBD. Further construction of models, molecular dynamic (MD) simulations and docking analysis were utilized to investigate the structure and composition of the N-glycans and their effects on HA activity.

## Materials and Methods

### Protein Sequence Data and Phylogenetic Analysis

A dataset of the protein sequences for H5N1 HA was retrieved from the National Center for Biotechnology Information (NCBI) flu database (http://www.ncbi.nlm.nih.gov/genomes/FLU) accessed in Sep. 2011 [28], containing approximately 330 human reports in 3602 sequences. An alignment of whole sequences was performed by ClustalW 2.0 (File S1) [29]. To investigate the distribution of N-glycosylation sites in different clades, a smaller dataset was used for further analysis with representative virus, including human-infecting viruses such as A/Hong Kong/486/1997, A/Hong Kong/213/2003, A/Indonesia/560H/2006 and A/Cambodia/V0219301/2011, as well as candidate vaccine viruses, such as A/Viet Nam/1203/2004, A/bar-headed goose/Qinghai/1A/2005 and A/Egypt/N03072/2010. Unrooted phylogenetic tree was constructed using the Neighbor-Joining method and the Poisson correction model in MEGA 5.05 [30]. The internal branching probabilities were determined by bootstrap analysis with 1,000 replicates.

### MD Simulations for HA Models

The crystal structure of the H5N1 HA (Protein Data Bank/PDB code: 3GBM) was used as the original coordinate file. Only the chain A and B in 3GBM, which represent HA1 and HA2, respectively, were extracted by PyMOL 0.99 and used as an HA monomer [31], [32]. This new HA monomer was denoted by the A/Viet Nam/1203/2004 strain (04VN). Based on the alignment analysis, a 03HK-RBD-like HA model was modified by S159N, T160A and N227S, replayed with the mutator 1.3 plugin of VMD [33], and denoted as 03HK.

**Figure 1:**
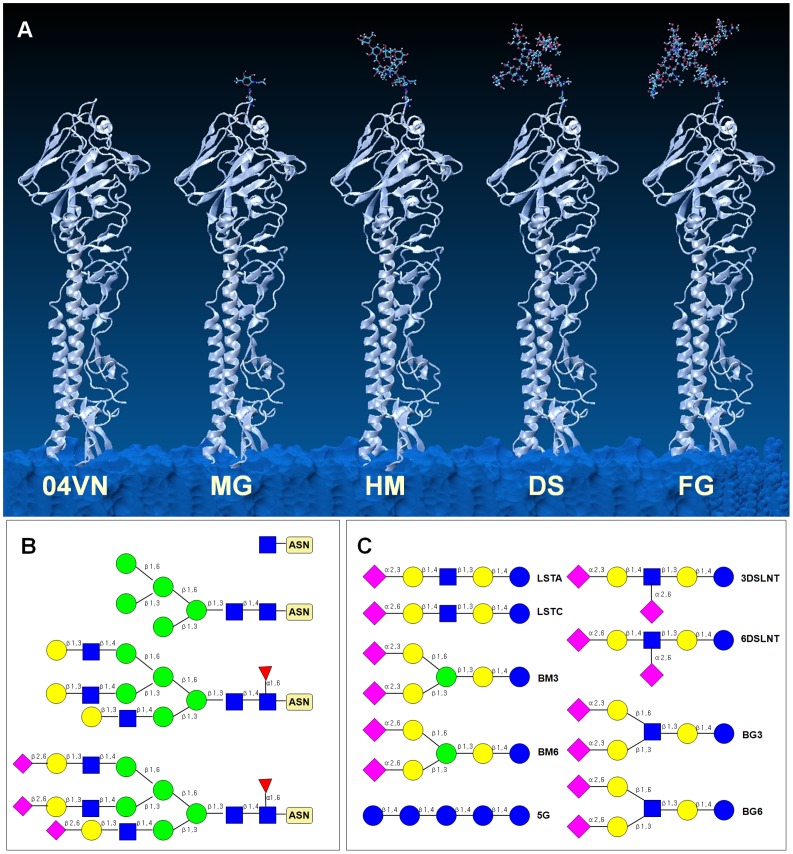
Schematic diagrams for HA and SA receptors models. (A) Five initial models of HA are represented by 04VN, MG, HM, DS and FG respectively. Only one glycan is added on 158N near the RBD. (B) Four glycans on glycosylated HAs are represented by MG, HM, DS and FG respectively. (C) Eight sialoglycans and one pentaglucose are used for docking assays. The abbreviations for each glycan are as follows: lactosialyltetraoses (LSTa/LSTc), disialyllacto-N-tetraose (3DSLNT/6DSLNT) and bisialyantennas based on two monosaccharides (bisialyantennary mannose, BM3/BM6 and bisialyantennary GlcNAc, BG3/BG6). The sequence of monosaccharides and glycosidic bonds are illustrated using Consortium for Functional Glycomics nomenclature.

To explore the influence of N-glycans near the RBD, four glycosylated HA models were constructed by the “GlycoProt” program (http://www.glycoscience.de) [34]. In consideration of existing studies, four glycans were added only at the site 158N in 04VN (Figure 1A and 1B). The names of four models described their glycan feature: mono N-acetyl-D-glucosamine HA (MG) had only one N-acetyl-D-glucosamine (GlcNAc) residue on 158N, high-mannosylation HA (HM) represented a mannose-rich glycan and the native full-glycosylation HA (FG) was commonly expressed from the mammalian cell system. Comparatively, the de-sialic acid HA (DS) model was a typical production treated with neuramidinase [26].

To perform the MD simulation, six HA models were solvated in rectangular water boxes, and the largest system had 70,000 atoms (including protein, glycan and waters) in a 140 Å×70 Å×70 Å cuboid of TIP3P water molecules. Minimization and equilibration were performed using the NAMD 2.8 program [35] with the CHARMM22 all-atom force field for the protein [36]; the missing parameters for the glycans were adopted from the GLYCAM06 and top_all36_carb V1.83 force field [37], [38]. A periodic boundary condition was used along with the particle mesh Ewald method for electrostatic interaction and a 12.5 Å cutoff for Van der Waals interaction. All of the systems were first energy-minimized for 0.5 ns with the protein and glycan atoms fixed and then for another 0.5 ns with all of the atoms relaxed. After the minimization, the system was heated from 0 to 300 K in 1 ns and then equilibrated for 4 ns with the pressure and temperature controlled (NVT). The temperature was held at 300 K using Langevin dynamics, and the pressure was held at 1 atm by the Langevin piston method. Production runs were subsequently performed for another 10 ns for each HA model.

The root-mean-square deviation (RMSD) was used to evaluate their stability, which was defined as the active range of the non-hydrogen atoms in the protein backbone and the pyranose ring in the glycans during the production runs. The root-mean-square fluctuation (RMSF) was used to compare the flexibility of the glycan residues and amino-acid residues. The RMSF reflects the fluctuation of the α-C atoms in amino-acid residues or the C2 atoms in glycans. The RMSF of the protein backbone was calculated by sampling every 2 ps, whereas the RMSD was extracted at 2 ps interval during the production runs [39].

### Docking Analysis of HAs and SA Receptor Complexes

To compare the interaction between HAs and SA receptors, four SA-α-2,3-Gal analogs and four SA-α-2,6-Gal analogs were designed and constructed to mimic the sialoglycans on human and avian cells. These SA receptors could be divided into four types: lactosialyltetraoses (LSTa/LSTc), disialyllacto-N-tetraose (3DSLNT/6DSLNT; 6DSLNT does not actually exist in humans) and bisialyantennary based on two monosaccharides (bisialyantennary mannose, BM3/BM6 and bisialyantennary GlcNAc, BG3/BG6). Additionally, a 5-glucose-pentose (5GLC) was used as a negative quality control (Figure 1C). All of the receptors were constructed by the SWEET2 program and optimized by the MM3 force field [40].

Six reasonable HA models gained after the 16 ns MD simulation were used for the flexible docking. Each model was docked with 9 receptors by turns. AutoDock 4.2 was used to perform the flexible docking in which the grid spacing was set to 0.375 Å and each grid map consisted of 80×80×80 grid points in three dimensions [41]. For each docking analysis, the grid center coordinates were set as the mean coordinates of the RBD. All of the glycosidic linkages from the receptors were treated as flexible linkages and allowed to rotate in 20-degree increments, together with key amino-acid residues in the RBD. With the other options set to the default values, 100 docking runs were performed during each docking experiment, and the results were analyzed by cluster analysis in their largest cluster. For each flexible docking, the three binding models with the lowest binding energies were selected for further statistical analysis. The flexible docking calculation and complementary analysis were performed on a Windows XP workstation.

### MD Simulations for Free Sialoglycans and Sialoglycan-HA Complexes

While the knowledge about topological structures of free or docked sialoglycans is limited, MD simulations make it possible to explore the adpoted characteristics. The explicitly solvated MD simulation of sialoglycans for 50 ns, together with 5 ns docked MD simulation in trimer HA have provided essential dynamic information about free sialoglycans conformations. The θ angle parameter, which define the angle between the C2 atom of SA1 and the C1 atoms of Gal2 and GlcNAc3 (or Man3), was used to describe the topologies adopted by various sialoglycans [42]. As the θ angle was maked up of the last three residues, it could reflect the dynamic changes during simulation complementally.

The initial conformations of 5 ns docked MD simulation were attained from optimal trimer sialoglycan-FG docking complexes by symmetry transformation script, then three complex N-glycans were added on 169N in the trimer docked complexes [33], [34]. For each trimer complexes, the MD simulation were performed through the same procedure as described previously.

The volumetric analysing method, which presented the overall spatial volume sampled by the glycans, used for monitoring the N-glycans and sialoglycans. As the powerful visual method to judge the interaction between the sialoglycans and N-glycans, the volumetric methods were constructed by averaging atomic density of the extracted MD frames using the VMD Volmap Tool plugin with 1 Å resolution grids [33].

All the MD simulations (NAMD_2.8b1_Linux-x86_64-CUDA) were performed on an ubuntu 10.04-based workstation, and the benchmark maintained 1 ns/day with the maximal system.

## Results

### Phylogenetic Analysis of Glycosylation Sites of HA in H5N1 Virus

There have been several works about the phylogenetic analysis in H5N1. The evolutionary dynamics of eleven genes in H5N1 virus are complicated, but the substitution rates of HA and neuramidinase (NA) are significantly higher than the rates of the internal protein segments (such as PA or M1 ) [43], [44]. According to a nomenclature system devised for H5N1 in 2008, the HAs can be divided into ten phylogenetic clades (0–9), of which three clades, namely clade 0, clade 1 and clade 2, are responsible for most human infections [45]. There was a marked divergence in the last four years, as several new subclades emerged and predominated in the H5N1 epidemics. When some groups in one monophyletic clade meet the divergence criteria (bootstrap values >60), they can split into second, third or even fourth-order clades [46]. The clade 2 began to spread westward during 2005; reached the Middle East, Europe and Africa in 2006; and caused hundreds of deaths within the past five years. Within clade 2, clades 2.1, 2.2 and 2.3 were responsible for most human cases in North Africa and Southeastern Asian. In addition, clades 2.4 and 2.5 had no relationship with human infections. Another major group, clade 1, which was isolated mainly in China, Cambodia, Thailand and Vietnam, was responsible for most documented human infections in Asia during 2004–2005, Thailand during 2006 and Cambodia during 2010–2012. Remarkably, after a 5-year quiet period, clade 1.1 reemerged in Cambodia with a 100% human fatality rate [4]. However, several previously circulating clades were not detected for the past 4 years, and it was likely that these clades were supplanted by the new clades. Based on an updated unified nomenclature system, the current circulating H5N1 virus in the past two years are centralized primarily in clades 1.1, 2.2.1, 2.2.1.1, 2.2.2, 2.3.2.1, 2.3.4, 2.3.4.2 and 7.1 [46].

**Figure 2:**
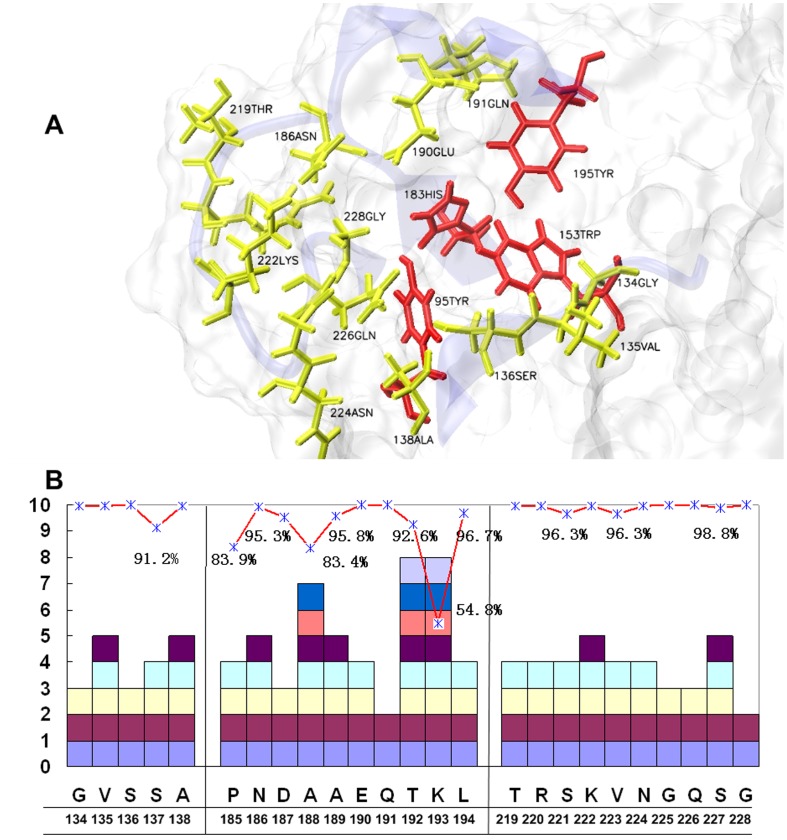
The amino-acid residues in the RBD of H5N1 HA. (A) The RBD in H5N1 HA consists of three secondary structure elements, Loop130, Loop220 and Helix190, together with four 100% conserved residues at the bottom (red). The residues with a conservation rate higher than 99% are labeled in yellow. (B) The statistics of the conserved amino-acid residues in the RBD. The predominant amino-acid residues are underlined, and the number of different types of mutations are shown as various blocks. Those with conservation rates lower than 99% are labeled. All of the residue numbers were adopted from the H3 HA numbering system.

The homology of the 3602 HA dataset was rather low, 25 out 325 amino-acid residues in HA1 revealed an identity rate of 100% (File S1). Those conserved residues were closely associated with the essential functions of HA; four residues located at the bottom of the RBD, 95Tyr, 153Trp, 183His and 195Trp exhibited an identity rate of 100%, and other residues in the RBD were also conservative relatively (Figure 2). One serious human isolate of H5N1 HA (A/Hong Kong/213/2003) had a single amino-acid residue difference in the RBD compared with most avian and human IVs. The S227N would result in an enhancement of its ability to bind to a human host [16]. Actually, since 2003, nine human records appeared in ten S227N mutations, such as the A/Cambodia/V0606311/2011 (File S1).

**Figure 3:**
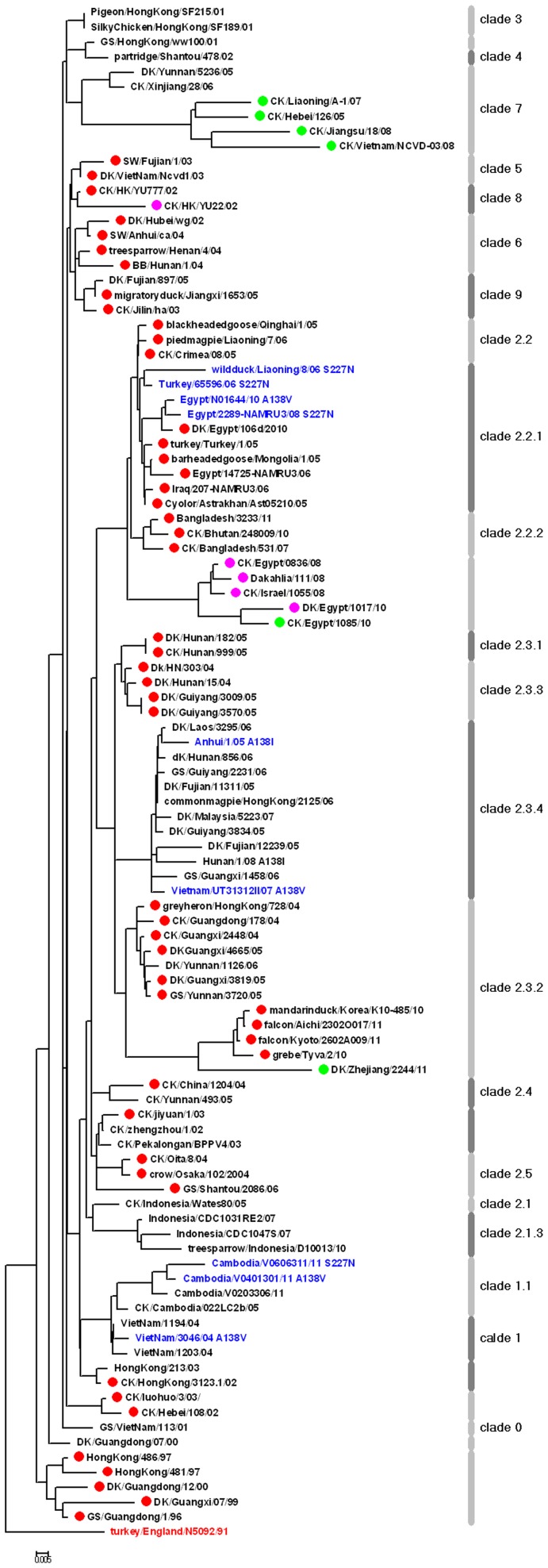
Phylogenetic tree of 103 HAs from H5N1 strains. Phylogenetic trees are inferred from protein sequences by the Neighbor-Joining method and rooted using A/turkey/England/1991(red text). Estimates of the statistical significance of the phylogenies are calculated by performing 1,000 bootstrap replicates. The lengths of the horizontal line are proportional to the numbers of protein sequence differences, as indicated by the scale bars. Different clades classified by the WHO are shown as grayish-white bars. Virus with glycosylation site 158N deficiency are labeled in red, whereas glycosylation site 169N deficiency are labeled in green, and the dual deficiencies are labeled in purple. Several important mutation strains mentioned in the article are labeled in blue.

As is known, N-X-T/S (X cannot be a proline) is the motif of an N-glycosylation site. The statistical analysis of 3,602 records elucidated that seven conservative glycosylation sites appeared in H5N1 HA. The conservation rates of glycosylation at 15N, 27N and 290N in HA1 and 154N and 213N in HA2 were 99.86%, 99.75%, 99.69%, 99.88%, and 99.73%, respectively (File S1). Another two glycosylation sites appear to be more complex. The conservation rate of glycosylation on 169N was 95.73%. All of the 169N glycosylation site deficiencies presented an avian specificity, and the earliest deficient record was A/Turkey/England/1991, the root of the tree in Figure 3. This glycosylation site was conserved until 2006; henceforth, sporadic deficiency cases have been reported in China, Vietnam and Egypt. Most of these cases were in clade 2.2.1 or clade 7.1. The conservation rate of the glycosylation on 158N was 47.61%. Strains with glycosylated and non-glycosylated 158N have co-existed extensively even before 1997. According to our phylogenetic analysis, viruses with 158N glycosylation site deficiency existed in nine clades extensively (all except clade 7, Figure 3). Most recent human records belonging to clade 2.2.1 in North Africa stayed a stable 158N glycosylation site deficiency. In contrast, the human virus belonging to clade 1.1 or clade 2.3.4 in Southeast Asia were conservative for the 158N glycosylation site. This discrepancy confirms that no close relationship exists between the loss of the 158N glycosylation site and host switching. Moreover, approximately 55 dual-glycosylation site (158N and 169N) deficiency virus were responsible for avian infections, most of which clustered in clade 2.2 during 2007 and 2008.

**Figure 4:**
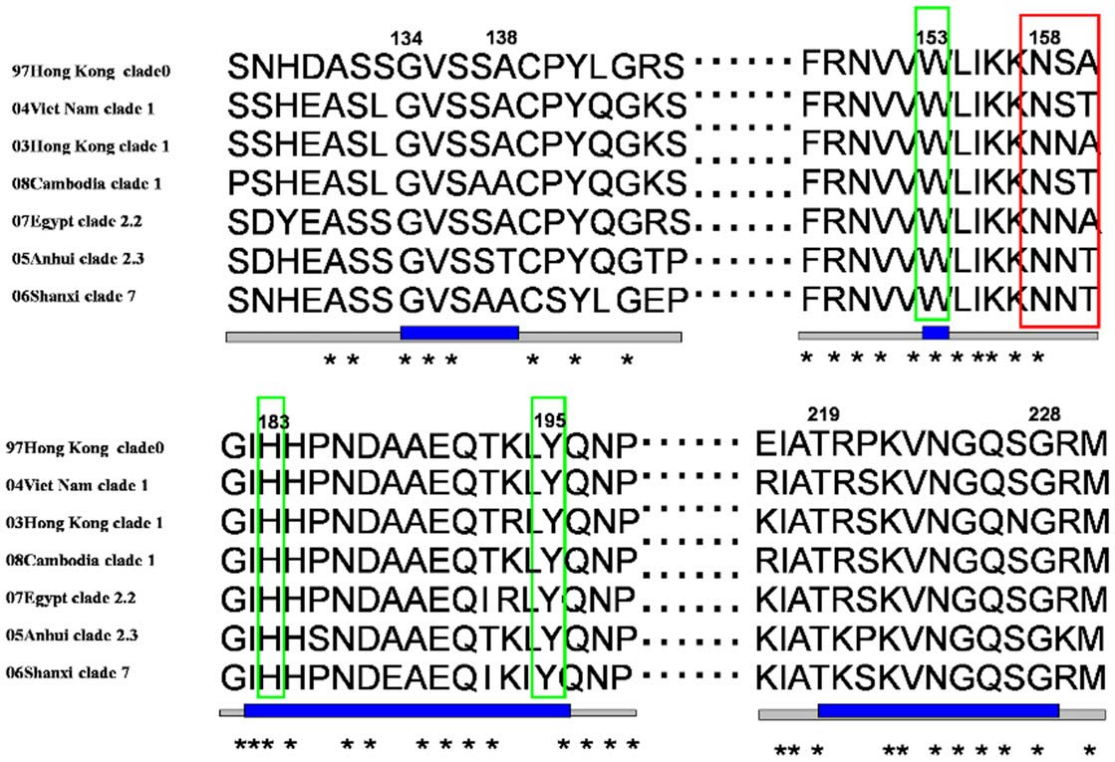
Sequence alignment of A/Hong Kong/486/97, A/Viet Nam/1203/2004(3GBM), A/Hong Kong/213/2003, A/Cambodia/S1211394/2008, A/Egypt/2321-NAMRU3/2007, A/Anhui/1/2005 and A/chicken/Shanxi/10/2006. The RBDs are shown in blue lines with the identical amino acids marked with asterisks. The glycosylation site 158N are boxed in red, whereas the four most conserved residues at the bottom of the RBD are boxed in green.

To confirm the evolutionary position of the HAs in the PDB and remodel the comparable HA models, further investigation was undertaken in H5N1 HAs from the PDB. Seven H5 HA structure files were available, of which three incomplete structures, 1JSO, 1JSM and 1JSN, represent the A/Duck/Singapore/3/97 monomer [47]. The files of 2FKO and 3GBM correspond to A/Viet Nam/1203/2004, whereas 2IBX and 3FKU represented A/Viet Nam/1194/2004 [48], [49], [50]. The similarity of the last four HA sequence was 98.27%, with the identical RBD (Figure 4). Accordingly, 3GBM would represent four HAs in subsequent experiments due to its minimal structural resolution. Although two strains mentioned above are responsible for human infections, Yamada *et al*. showed that A/Viet Nam/1194/2004 still had a high affinity for SA-α-2,3-Gal receptors [49]. Coincidentally, A/Viet Nam/1194/2004 and A/Viet Nam/1203/2004 belong to clade 1 and both are chosen as candidate vaccine viruses. A/Hong Kong/213/2003 is another definite human virus that also belongs to clade 1. As Figure 4 showed, the RBDs of various H5N1 clades were highly conserved, the only differences among the three strains were residue 227 and the glycosylation site at 158N. A T160A mutation in “158NST” would result in the deficiency of this glycosylated site.

### MD Simulation for HA Models Reveals Stable RBD and Relative Flexible Glycans

Glycosylation exerts a significant influence on envelope glycoproteins. To perform the MD simulation for glycoprotein, the complete coordinate file is necessary. Unfortunately, complete larger glycans are too flexible to yield sufficient electron density; consequently, their 3D structures cannot be derived using X-ray crystallography. Many oligosaccharide NOEs (Nuclear Overhouser Effects) also cannot be resolved and are difficult to assign by NMR. Additionally, there are often too few inter-residue NOEs to render an unambiguous 3D structure determination possible [51]. Given this situation, glycosylated HAs must be reconstructed manually.

**Figure 5:**
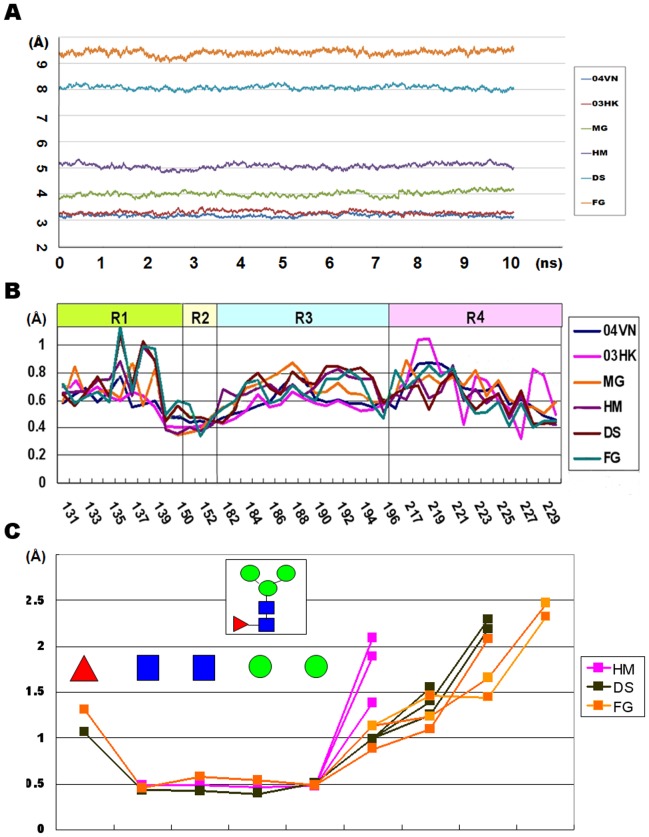
The dynamical properties of the HA models during MD simulation. (A) Six convergent RMSDs of the HA models indicate the stable states, while highly glycosylated HAs experienced larger fluctuation against the initial conformations. (B) The RMSF values reflect the fluctuation of those residues in the RBD. The S227N mutation in 03HK would result in a relatively stable 222Lys (with a lower RMSF value in 222). The glycans at 158N of affect their acroteric amino-acid residues. (C) The RMSF of the three glycans on HM, DS, and FG present the complicated praxiology that the glycan residues in the core are more stable compared with the terminal glycan residues. Columns 2–5 represent the RMSF in the core of the N-glycan compared with the glycan residues at the branches and terminus.

The individual RMSFs in the RBD showed significant difference. Those relatively active residues, which have a higher RMSF and less impacted by steric clashes, may change their conformation to accommodate the receptors. During the 16 ns MD simulations, the energetic and structural properties were monitored for the 6 systems. The RMSDs and the energies converged in all of the systems, which indicated well-behaved simulations (Figure 5A). We found that the helical structure and the overall protein backbones were stable during the whole equilibrium, whereas the loop experienced larger fluctuations relatively. Loop220 may fluctuate more than Loop130 and Helix190, based on their respective RMSFs. Considering the results from the phylogenetic analysis, four most conserved residues, 95Tyr, 153Trp, 183His and 195Tyr, located at the bottom of the RBD, had lower RMSFs compare with the non-conserved residues (Figure 5B). By analyzing the MD trajectories, the replaced residues in 03HK would affect their acroteric amino-acid residues. The RMSF of 222Lys in 03HK decreased, which was responsible for the interaction of the basic group in 222Lys with the newly introduced, weakly acidic amide group of the S227N residue, a motion that made the two sides of Loop220 become closer. Moreover, by changing S159N and T160A in 03HK, the RMSFs of the adjacent residues were also changed, which indicated the steric alteration.

The behavior of N-glycans on HAs was also investigated. The RMSDs of the longer glycans in HM, DS and FG had similar features: at the minimization stage, the RMSDs rose vigorously; however, the whole glycan became stable and convergent immediately. Each monosaccharide in the N-glycan had a corresponding RMSF that reflects its activity, such as the mannoses in HM, GalNAc in DS and SA in FG; as was shown in Figure 5C, the higher RMSFs in the distal monosaccharide residues revealed their active fluctuation.

### Energy Analysis from HAs and SA Receptor Docking Complexes

The interaction between HA and SA receptors could be deemed as a kind of docking mechanism. Several software programs for molecular docking analysis have been developed in recent years, such as Dock and AutoDock [52], [41]. AutoDock 4.2 adopts the Lamarckian genetic algorithm to estimate the binding energies and explore the most probable docking conformation, of which the docking conformation with the minimal value may be closely related to a natural state.

**Figure 6:**
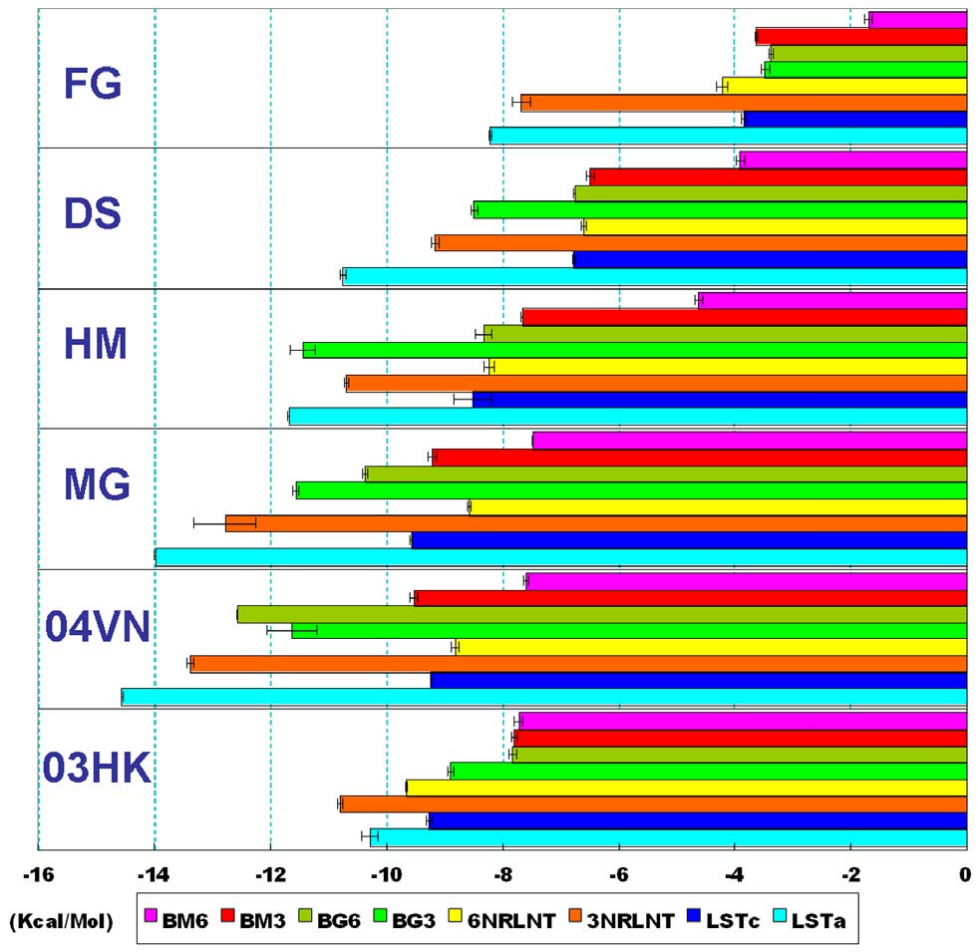
The binding energies collected from flexible docking. The binding energies are calculated from the three lowest energies provided in the largest clusters. A notable tendency is the increasing binding energy accompanying with increasing glycosylation.

Based on the binding energies provided from the output files, all HAs showed a conspicuous affinity for α-2,3-sialoglycans or α-2,6-sialoglycans (Figure 6). Interestingly, as the negative quality control, the binding energy from HA-5GLC complexes presented a positive value of approximately +3- +6 kcal/mol, which corresponded to a nonbinding property between HA and glucoses. In the docking assays, all the HAs presented relatively lower energies with α-2,3-sialoglycans and higher values with α-2,6-sialoglycans, which indicated that the HA of A/Viet Nam/1203/2004 would still have a preference for avian sialoglycans, although 04VN was derived from a human infection. One explanation for this paradox could be that humans exposed to a high concentration of A/Viet Nam/1203/2004 strains would also be infected [49].

A notable tendency was the increasing binding energy accompanying increasing glycosylation, which demonstrated that the binding affinities were impaired by complicated glycans. The average binding energies collected from the glycosylated HAs were higher than those from 04VN and 03HK.

Another remarkable feature in the 03HK-sialoglycans docked complexes which puzzled us, was that there is no obvious numerical difference between the two types of sialoglycan docking assays (Figure 6). Although a standard error of approximately 2–3 kcal/mol in predicted binding energies would be acceptable [41], we supposed that the existing amino-acid residue mutations in A/Hong Kong/213/2003 did not enhance the binding affinity to α-2,6-sialoglycans but decreased its ability to bind α-2,3-sialoglycans. An analogical description in H3N2 HA noted that the trans conformation of SA-α-2,3-Gal directs the Gal ring away from the receptor-binding domain surface, and the amide group of Gln226 formed an H-bond with the axial 4-hydroxyl group of the penultimate Gal [27]. In this case, the total binding energy would be impaired by the 227Ser with a hydroxyl group being replaced by the Asn with a larger amide group.

### Conformation Analysis from HAs and SA Receptor Docking Complexes

Carboxylate, hydroxyl, N-acetyl and glycerol group are four functional groups in SA residue that participate in numerous hydrogen bonds (H-bonds) and hydrophobic interactions with the RBD. Those H-bonds formed from the hydroxyl groups in the penultimate Gal and Loop220 are responsible for host preference [53].

**Figure 7:**
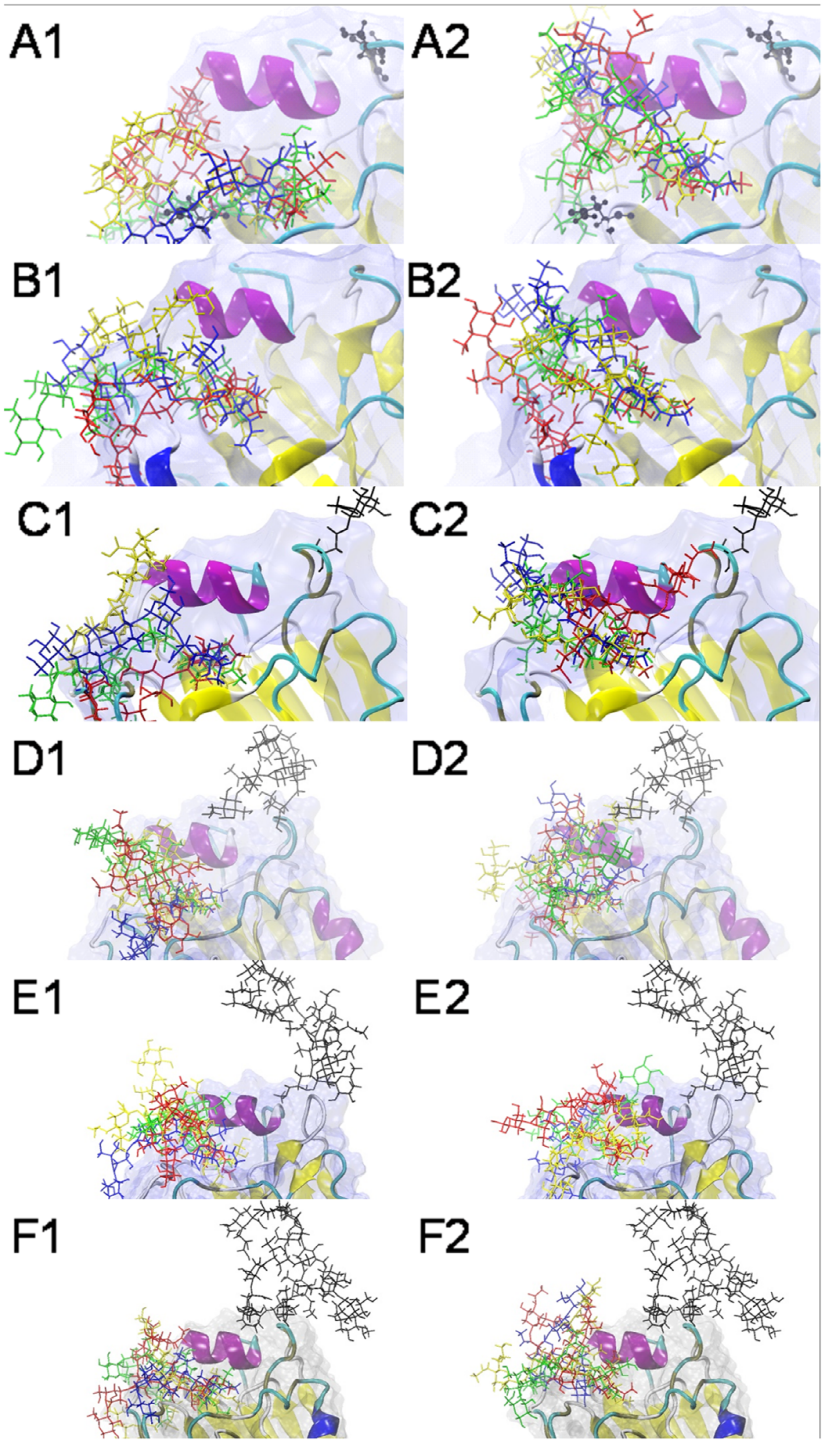
The docking complexes between six HAs and eight sialoglycans. SA-α-2,3-Gal receptors are superimposed with distal SA residues at left, whereas the SA-α-2,6-Gal receptors are superimposed with distal SA residues at right. LSTa/LSTc are shown in blue, 3DSLNT/6DSLNT are shown in green, BM3/BM6 are shown in red and BG3/BG6 are shown in yellow. The types of receptors deploy distinctive topologies: most SA-α-2,3-Gal receptors are straight and extrorse, whereas the SA-α-2,6-Gal receptors are fishhook-like and ental. (A) 03HK-sialoglycan docking complexes, (B) 04VN-sialoglycan docking complexes, (C) MG-sialoglycan docking complexes, (D) HM-sialoglycan docking complexes, (E) DS-sialoglycan docking complexes, (F) FG-sialoglycan docking complexes.

Commonly, the SA in α-2,3-sialoglycans and α-2,6-sialoglycans adopt the trans or cis conformation, respectively, which is oriented to the 158N glycosylation site [12], [27]. In all of the docking complexes, the carboxylate, hydroxyl, N-acetyl and glycerol groups in SA buried inside the shallow pocket (Figure 7). This reflected that HA, as a kind of glycan-binding protein (GBP), had a natural tendency to bind SA. The penultimate Gal were distinctive based on the preference of HA, as not all of the Gals would fit into the RBD due to the steric clashes of amino-acid residues or N-glycan, much less the antepenultimate GlcNAc/Man in receptors. Most antepenultimate residues would form a β1-3 or β1-4 glycosidic linkage with the penultimate β-D-Gal. As a result, the α-2,3-sialoglycan and α-2,6-sialoglycan conformations were predominantly straight and fishhook-like, respectively (Figure S1, S2). The topologies of the α-2,3-sialoglycans was extrorse, whereas the α-2,6-sialoglycans, which lean toward Helix190, were ental.

As the long glycans, LSTa and LSTc would interact with the RBD in a pre-description [12]. Due to the fishhook-like topology, the remainder of the residues in LSTc exhibited more contact with Helix190 compared with LSTa. The branched 3DSLNT is the most abundant sialyl human milk oligosaccharide; in contrast, 6DSLNT does not exist in human milk [54]. Another α-2,6 glycosidic linkage was formed between a second SA and a GlcNAc, however, this α-2,6-linked SA could not fit into the RBD. Although there were two SA-α-2,6-Gal or SA-α-2,3-Gal linkages in biantennary sialoglycans, these did not enhance the binding affinity. Subsequent conformation analysis showed that only the SA in one antenna would fit into the RBD, whereas the remaining components interacted slightly with several residues. Actually, one biantennary sialoglycan molecule cannot dock with two RBDs in a trimer HAs due to the short distance between the two antennas. The estimated curvilinear distance between two RBDs is approximately 60 Å, whereas the maximal distances between two SAs in most bi- or tri-antennary sialoglycans are shorter (30–40 Å).

The SA receptors would interact with the RBD through two forces:hydrogen bond (H-bond) and hydrophobic interaction. H-bond was the essential interaction in stabilizing ligand-receptor complexes. There were copious polar groups in the receptors and RBD that facilitate hydrogen-bond formation. During the course of dynamic bonding, several persistent and transient hydrogen bonds could be observed: most persistent H-bonds were formed from the hydroxyl groups offered by SA and Gal with the side-chain groups. In all of the docking complexes, the four most conserved residues located at the bottom of the RBD have formed stable H-bonds with SA: the glycerol group in SA interacted with the p-hydroxyphenyl group of 195Tyr, the imidazole group of 183His interacted with the glycerol group of SA and the carboxyl group in SA formed a stable H-bond with the p-hydroxyphenyl group of 95Tyr. Obviously, Loop220 played an important part in the formation of H-bonds with the penultimate Gal. Many observable transient H-bonds were formed from the remainder of sialoglycans and non-conserved residues around the RBD. One typical example was the residue 194Leu or 190Glu, located in Helix190, which would form an H-bond with the antepenultimate GlcNAc/Man, especially in α-2,6-sialoglycans. In various hydrophobic interactions, one common stacking force was formed by the N-acetyl group in SA and 153Trp, whereas other aromatic groups are far from the docking pocket. Several residues, such as Ser226, Trp153, His183, or Gln191, would also form hydrophobic interactions with sialoglycans, as in the previous descriptions [27].

The Ser has a hydroxy group at the γ-C atom that can form an H-bond with other polar groups, such as water or the receptor. In the docking conformations collected previously, however, the residue 227Ser did not participate directly in stabilizing interactions with the penultimate Gal. A persistent H-bond between 226Gln and Gal formed in several HA-α-2,3-sialoglycan docking complexes but rarely in HA-α-2,6-sialoglycans docking complexes [12]. After the introduction of the S227N mutation, an amide group would interact with the adjacent basic group in 222Lys, forming a more compact Loop220, which moved 226Gln away from its previous position. This change would decrease the probability of the above H-bond formation, which caused most S227N strains exhibiting a decreased affinity for α-2,3-sialoglycans. Moreover, almost all S227N mutations are followed by the loss of the 158N glycosylation site (File S1), the deficiency of this glycosylation site is conducive to bind the α-2,6-sialoglycans. Hence this slight change may be one reason responsible for more human infections [26].

We found that all of the glycosylated HAs except MG exhibited a clearly weakened binding ability. One factor was the steric clashes that would block the complex, bi- or tri-antennary sialoglycans to the target proteins. The topologies of glycans, the distribution of hydroxy groups in different saccharide residues, the amide group in GalNAc and GlcNAc or the groups in SA would also affect the binding affinity. Analysis of binding energies and conformations indicated that N-glycans on 158N have more influence on fishhook-like α-2,6-sialoglycans.

### Glycosylation as a Subsidiary Contribution in Receptor Recognition

Several epitope sites in the tip of HA1 are near the RBD, and one function of glycosylation is to provide the shield to escape the host immune system [55], [56]. As a type of hydrophilic long-chain and high-mass molecule, N-glycans have only one covalent bond to the Asn, which discloses that the N-glycans are rather mobile and flexible. Undoubtedly, the presence of huge N-glycans would impact the binding efficacy of the RBD.

One factor that cannot be neglected in trimeric HAs is that some glycosylation sites appear to be far from one RBD (>25 Å), but their glycans still affect the binding affinity for the adjoining RBD in another monomer. Such as the 169N glycosylation site: the distance between the center of the RBD in chain A and 169N is 34.18 Å, whereas the distance to 158N is 18.72 Å; however, it would be 20.05 Å between the RBD and the adjoining 169N (measured by the nitrogen atom in Asn and the weight center of the RBD, from a trimeric HA coordinate file coded 2IBX). A previous survey showed that the conservation rate of the 169N glycosylation site is 95.73%, whereas an S171A change would result in the loss of this glycosylation site (File S1).

**Figure 8:**
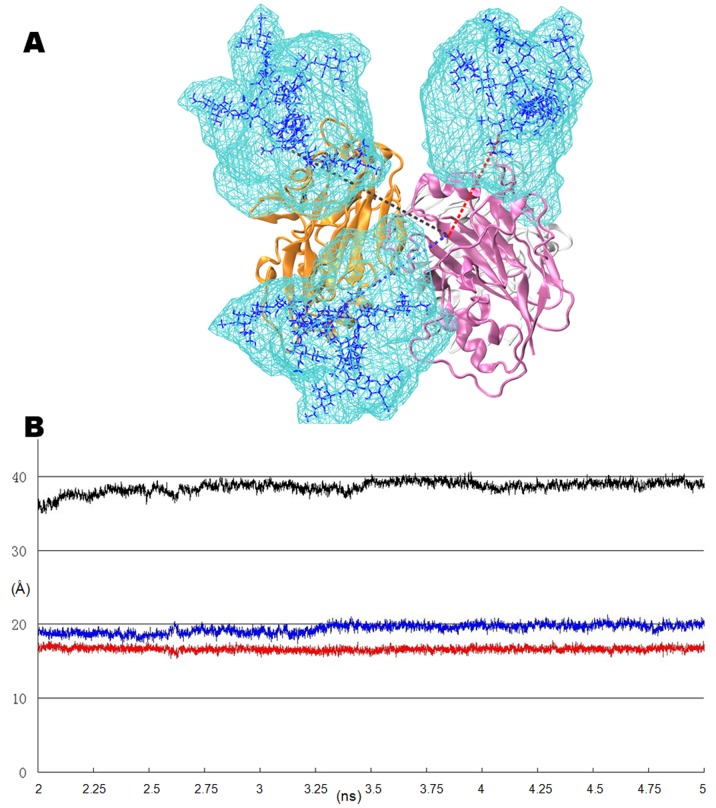
The influence of vicinal N-glycans on the RBD of HA trimer. (A) The volumetric topologies of the N-glycans near the RBD during the 5 ns MD simulation. The complex N-glycans on the sites 158N and 169N would swing dramatically. (B) The distances from the weight center of the RBD to the three topological centers of the N-glycans are calculated at 10 ps intervals, three colors represent the corresponding distances in (A).

As observed from the trimer HA in Figure 8, we found that one RBD in chain A was located among three glycosylated sites: 158N in chain A and 169N in chain E, with the distance from the center of the RBD to the center of each glycan maintained at approximately 18 to 21 Å. Additionally, the distance between the center of the RBD and the third glycosylated site, 158N in chain E, is approximately 35–40 Å. As summarized from the MD simulation and docking complexes, the SA-α-2,3-Gal and SA-α-2,6-Gal receptors deployed two distinctive topologies (Figure S1, S2 and S3). It could be hypothesized that the shapes and components of the glycans on HA have various influences: huge glycans around the RBD would restrict the affinity and specificity of HA.

The N-glycans and sialoglycans topologies were examined using the volumes sampled. The orientations and shapes of the volumetric maps varied dramatically for all the glycans: three N-glycans on 158N and 169N were swinging dramatically while the sialoglycans constrained in the RBD, especially for the SA residues. Straight and extrorse topologies were observed in the free and docked SA-α-2,3-Gal receptors. Bound SA-α-2,6-Gal receptors adopted tightly folded and fishhook-like conformations and bented toward the Helix190. These two distinctive conformations resulted in different preference of contaction to the N-glycans on 158N or 169N (Figure S1, S2 and S3). Obviously, the loss of the 169N glycosylation site has an advantage in binding the straight and extrorse α-2,3-sialoglycans, whereas the loss of the 158N glycosylation site facilitates the recognition of the fishhook-like and ental α-2,6-sialoglycans. With more complicated glycosylation, the reasonable sialoglycans for docking would be more restricted.

## Discussion

There is one specified group of glycoproteins appearing on viral envelope that participates in the recognition and invasion, such as HA in IVs, gp160 in HIV and the spike (S) glycoprotein in the SARS coronaviruses [57], [58]. At the very first stage, these proteins recognize a specified glycan pattern on the surface of the host cell; from this perspective, these glycoproteins can be deemed as a kind of lectin or GBP. Many viruses target the distal SA, including Simian Virus 40 (SV40) and murine polyomavirus (Polyoma) [59], [60].

HA have a high specificity in host recognition. The mechanism in H1N1 HA is relatively clear: outbreak and epidemic HAs have a high preference for SA-α-2,6-Gal, which is distinctive in humans compared with SA-α-2,3-Gal in avian hosts. The E190D and G225D mutations in the RBD of H1N1 would result in host switching [61]. Similarly, the Q226L and G228S mutations in H2N2 and H3N2 would also lead to host switching. However, H5N1, as one of the highly pathogenic avian IVs, has not been reported to have such definite mutations until now. In the past five years, several subclades have predominated in the H5N1 virus, and some subclades exhibited a preference for human infection and were responsible for a large number of human deaths. In 2011, a 100% human fatality rate of clade 1.1 in Cambodia caused a great panic; however, genetic and *in vitro* binding analysis indicated that the receptor specificity of the H5N1 virus is still of the avian type, which suggested that the restriction by receptor specificity may not be as stringent as previously thought in certain avian viruses [61].

Though there are not sufficient cumulative data to analyze H5N1 compared with H1N1 or H3N2, prospective work can be performed by computer simulation. One common method is to explore the relationship between host switching and protein sequences. However, secondary sequences are not enough to further investigate the reaction mechanism. By conformation analysis of a three-dimensional structure, a concrete target can be found readily. The RBD in H5N1 HA mainly consisted of three conserved secondary structures; moreover, acroteric modifications, such as glycosylation, would also affect the binding preference. Conformation analysis showed that the acquisition or deficiency of an N-glycosylation site would be more effective than simple amino-acid residue mutations. Furthermore, the composition and shapes of the N-glycans on HA are dependent on the host N-glycan synthetic system in endoplasmic reticulum. Thus, the modification of heterogeneous N-glycans make the HA more intricate [21].

In this study, we presented a series of methods and models to simulate the effect of glycosylation on receptor binding *in silico*. The previous conclusion revealed that the RBD pocket of HA in H5N1 was shallow and specified; one terminal residue, SA, could fit into the RBD easily in different HAs. Detailed and complete studies have been conducted with several methods, and the mechanism of host switching and praxiology of HAs was clear. No covalent bonds existed between HA and its receptors, but the abundant H-bonds and hydrophobic bonds facilitated the docking conformation. A re-constructed 03HK-RBD-like HA model revealed that 03HK has a negative influence on the binding of SA-α-2,3-Gal. This change could be one reason that 03HK has a dual-host affinity. As mentioned previously, the 04VN model derived from A/Viet Nam/1203/2004, a vaccine candidate for humans, but still had a higher affinity SA-α-2,3-Gal.

Docking assays from four glycosylated HAs revealed similar outcomes to that of 04VN, but with numerical disparities. Generally, the HA models with increasing glycans have a gradually decreasing binding ability; larger glycans on 158N in the prominent antigenic loop on the edge of the RBD would obviously decrease the binding affinity for the SA-α-2,6-Gal analogs. Recent H5N1 viruses (especially in clade 2.2), which are characterized by a deficiency of the 158N glycosylation site, exhibit an increased propensity for acquiring human receptor specificity. This effect could be due to the large glycans on 158N, which would hamper the affinity for the fishhook-like α-2,6-sialoglycans, based on our results.

A typical monomer affinity in GBP is measured at the millimolar level, which is quite weak compared with enzymatic reactions, so-called “high affinity but low avidity”. To achieve the high avidity, like other GBPs, a complete HA consists with three monomers. The topologies of the three RBDs in the HA trimer face to three symmetry directions would facilitate the binding of the host SA glycans. As the primary interaction occurs in the RBD and SA and Gal, the remainder of the components in sialoglycans would also interact with the acroteric amino-acid residues. It is often overlooked that glycosylation at 169N of an adjacent monomer would also affect the binding affinity, especially for the straight SA-α-2,3-Gal analogs. Coincidentally, the deficiency of the 169N glycosylation site only appears in avian infection virus. The result showed that the N-glycans on two glycosylation sites would form the extensive steric clashes and restrict receptor specificity.

Actually, the factors for cross-species and human-to-human transmission in IVs are a composite mechanism. The preference switching of HA is only one necessary step; another envelope glycoprotein, NA, the function of which is related to the releasing of the virus, also participates in human infections. A definite human infection would be affected by virus, host and environment. To prevent a potential human epidemic caused by H5N1 virus, more investigations must be performed.

## Supporting Information

Figure S1 **The superimpositions of sialoglycans during 50** **ns explicitly solvated MD simulation reflect the free** S**A-α-2,3-Gal and SA-α-2,6-Gal receptors adopted two distinctive topologies.** (A) As the initial sialyglacans are shown as the cyan CPK models, nine representative conformations of sialoglycan from 5 ns intervals are superimposed with distal SA residues. It clearly revealed the remainder of all SA-α-2,3-Gal receptors are leftward, compare to the rightward SA-α-2,6-Gal receptors, which result in two orientations.
(TIF)

Figure S2 **Topological θ angle plots of the free sialoglycans during 50 ns.** (A) the θ angle in four SA-α-2,3-Gal receptors swinging between 110° and 180°. (B) the θ angle in four SA-α-2,6-Gal receptors swinging between 60° and 110°.
(TIF)

Figure S3 **The volumetric topologies of glycans in the Sialoglycans and trimer HA complexes.** As it indicated that the θ angles of sialoglycans are stable during 5 ns MD simultion in the trimer HA, all the SA-α-2,3-Gal receptors maintain a straight-like topology (110°<θ<180°) and SA-α-2,6-Gal receptors maintain a fishhook-like topology (60°<θ<110°) resepectively. The orientations and shapes of the volumetric maps vary dramatically for the N-glycans on 158N and 169N while the sialoglycans varied in the smaller spatial volume in RBD. More complicated N-glycans would sterically hinder the receptor binding, even with different preference.
(TIF)

File S1 **The alignment file containing the 3,602 protein sequences of H5N1 HA1.** As is shown in the file, the glycosylation sites are marked in red, whereas the 100% conserved residues are marked in cyan.
(XLS)
