# Supplementary figures and images for: Two Glycosylation Sites in H5N1 Influenza Virus Hemagglutinin That Affect Binding Preference by Computer-Based Analysis
Source: PLoS One. 2012 Jun 14;7(6):e38794. doi: 10.1371/journal.pone.0038794 (PMC3375263; doi:10.1371/journal.pone.0038794)

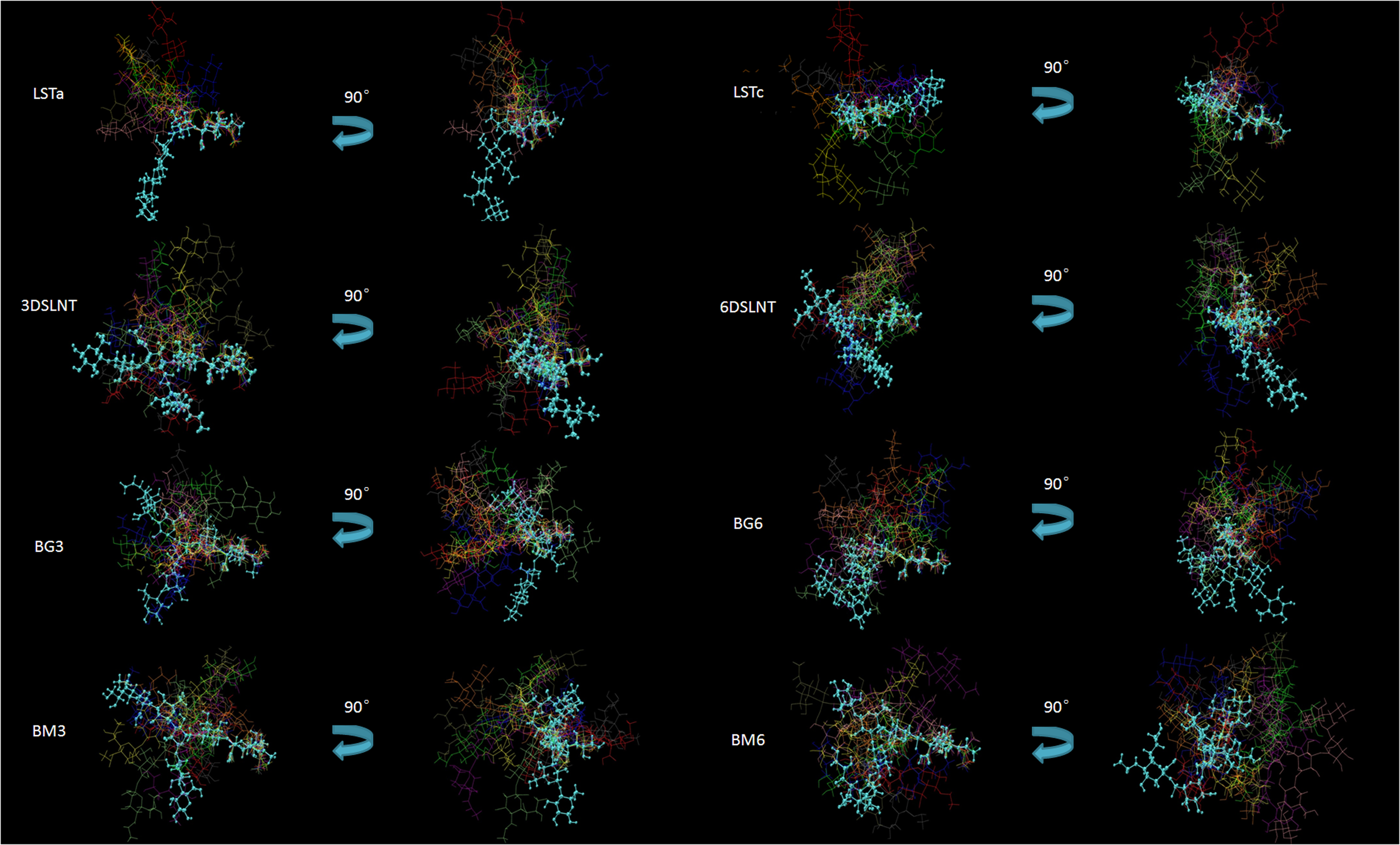

Supplement: Figure S1 — The superimpositions of sialoglycans during 50 ns explicitly solvated MD simulation reflect the free SA-α-2,3-Gal and SA-α-2,6-Gal receptors adopted two distinctive topologies. (A) As the initial sialyglacans are shown as the cyan CPK models, nine representative conformations of sialoglycan from 5 ns intervals are superimposed with distal SA residues. It clearly revealed the remainder of all SA-α-2,3-Gal receptors are leftward, compare to the rightward SA-α-2,6-Gal receptors, which result in two orientations. (TIF) [file pone.0038794.s001.tif]

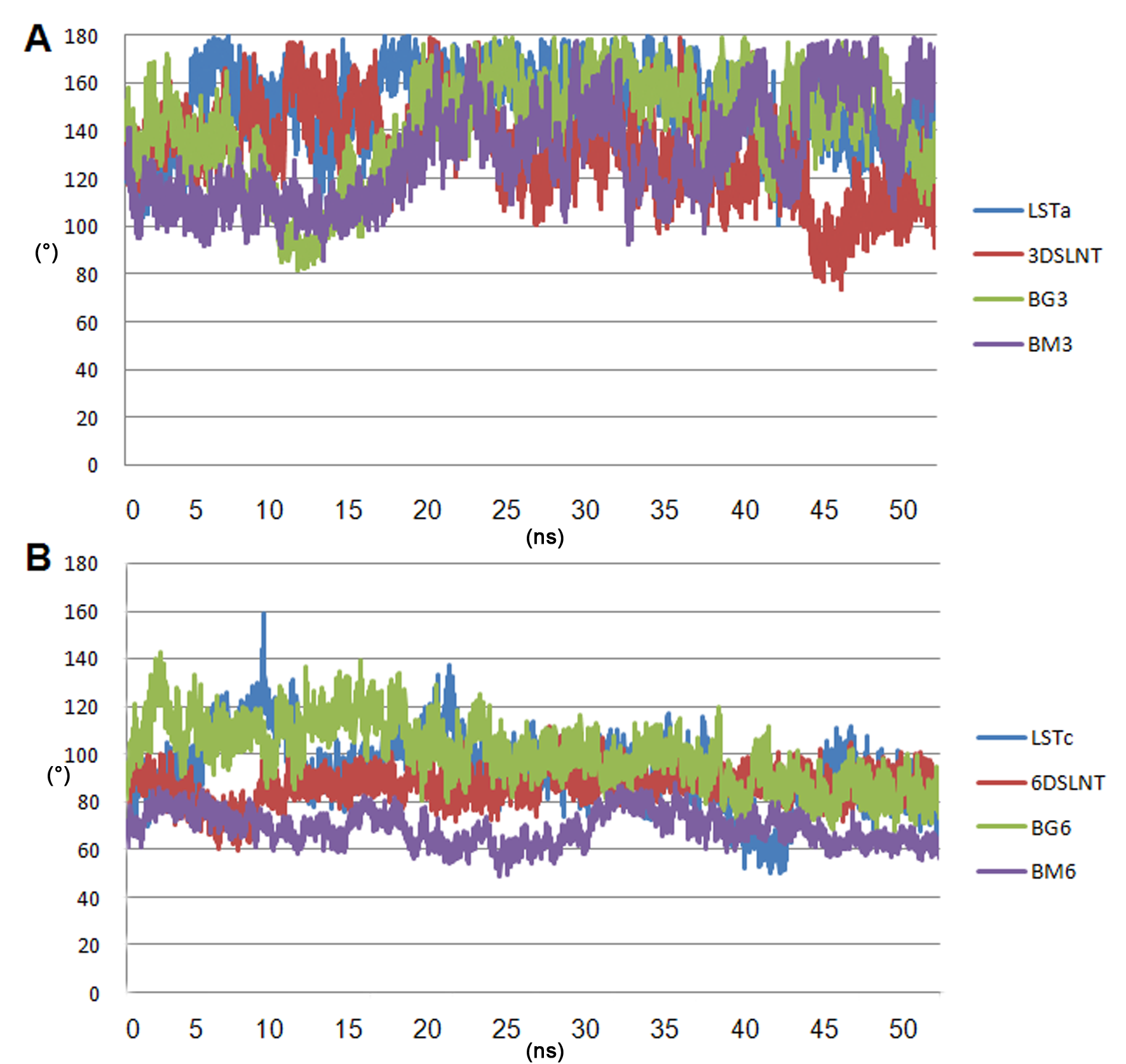

Supplement: Figure S2 — Topological θ angle plots of the free sialoglycans during 50 ns. (A) the θ angle in four SA-α-2,3-Gal receptors swinging between 110° and 180°. (B) the θ angle in four SA-α-2,6-Gal receptors swinging between 60° and 110°. (TIF) [file pone.0038794.s002.tif]

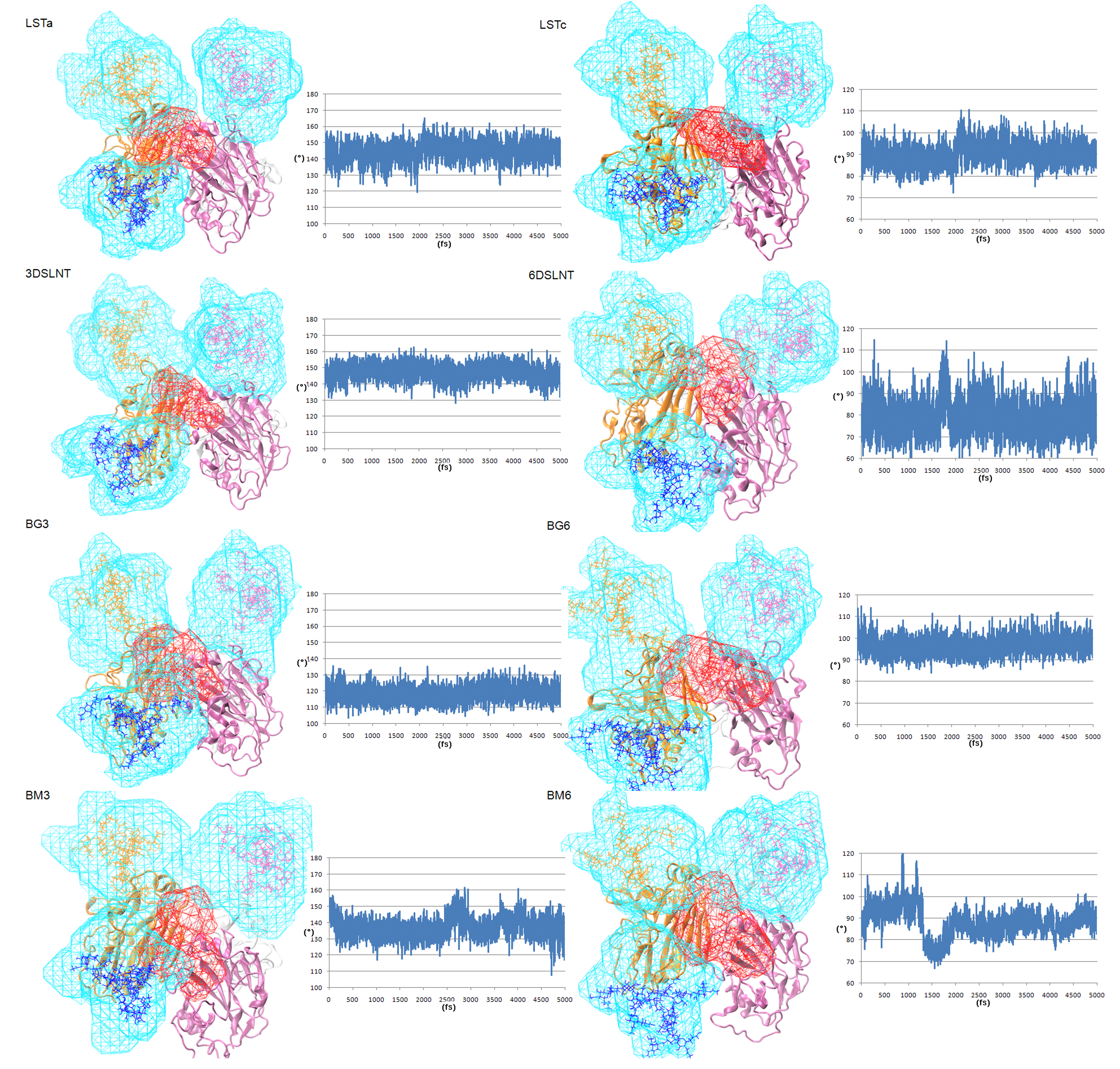

Supplement: Figure S3 — The volumetric topologies of glycans in the Sialoglycans and trimer HA complexes. As it indicated that the θ angles of sialoglycans are stable during 5 ns MD simultion in the trimer HA, all the SA-α-2,3-Gal receptors maintain a straight-like topology (110°<θ<180°) and SA-α-2,6-Gal receptors maintain a fishhook-like topology (60°<θ<110°) resepectively. The orientations and shapes of the volumetric maps vary dramatically for the N-glycans on 158N and 169N while the sialoglycans varied in the smaller spatial volume in RBD. More complicated N-glycans would sterically hinder the receptor binding, even with different preference. (TIF) [file pone.0038794.s003.tif]
